# Clinically refined epidemiology of nontuberculous mycobacterial pulmonary disease in South Korea: overestimation when relying only on diagnostic codes

**DOI:** 10.1186/s12890-022-01993-1

**Published:** 2022-05-13

**Authors:** Jae Hyeon Park, Sue Shin, Taek Soo Kim, Hyunwoong Park

**Affiliations:** 1 Department of Laboratory Medicine, Seoul National University Hospital, Seoul, South Korea; 2 Department of Laboratory Medicine, Seoul National University College of Medicine, Seoul, South Korea; 3 Department of Laboratory Medicine, Seoul National University Boramae Medical Center, Boramaro 5 gil 20, Dongjak-gu, Seoul, 07061 South Korea

**Keywords:** Nontuberculous mycobacterial infections, Insurance claim, Incidence, Prevalence, Clinical laboratory test

## Abstract

**Background:**

There have been reports of increases in the incidence and prevalence of nontuberculous mycobacterial pulmonary disease (NTM-PD) in several countries, but no studies have analyzed claims data using laboratory tests. This study aimed to estimate the nationwide epidemiology and medical treatments of NTM-PD according to laboratory tests run in Korea.

**Methods:**

Using claims data from the Health Insurance Review and Assessment Service, we analyzed patients with nontuberculous mycobacterium (ICD-10: A31) who were diagnosed from Jan 2007 to Jun 2019. The incidence and prevalence of NTM-PD and whether related laboratory tests were performed were analyzed. Diagnostic code-based NTM-PD patients were defined as patients who had NTM as a diagnosis on at least 2 occasions within 180 days. Clinically refined NTM-PD patients were defined as those excluding hospital-diagnosed patients with acid-fast bacilli (AFB) culture rates less than 5%. Laboratory tests included AFB smears, AFB culture, NTM identification, and drug susceptibility tests (DSTs).

**Results:**

A total of 60,071 diagnostic code-based NTM-PD patients were evaluated. Clinically refined NTM-PD included 45,321 patients, excluding 14,750 (24.6%) patients diagnosed in hospitals with low AFB culture rates. The annual incidence per 100,000 population increased from 2.9 cases in 2008 to 12.3 cases in 2018. The annual prevalence per 100,000 population increased from 5.3 cases in 2008 to 41.7 cases in 2018. After removing outliers according to the AFB culture rate, a significant decrease in incidence was observed in women younger than 50 years. Among patients with clinically refined NTM-PD, the test rates for AFB culture, NTM identification, and DST were 84.3%, 59.1%, and 40.4%, respectively. From the outpatient clinic, 17,977 (39.7%) patients were prescribed drugs related to NTM treatment, with a median number of prescriptions of 7 (interquartile range (IQR) 3–11) and a median duration from the diagnosis to end of treatment of 330 (IQR 118–578) days.

**Conclusions:**

Although the incidence and prevalence of NTM-PD are on the rise, the recent surge in women 50 years of age is overestimated in patients not adequately tested. In claim-based studies, there may be limitations in estimating the epidemiological data with only the diagnostic codes.

**Supplementary Information:**

The online version contains supplementary material available at 10.1186/s12890-022-01993-1.

## Background

Nontuberculous mycobacteria (NTM) are various bacteria in the genus *Mycobacterium*, except *M. tuberculosis* complex (MTB) and *M. leprae*, and they exist in environments such as tap water, natural water, indoor dust, and soil [1, 2]. Over 200 species of NTM have been identified to date, but only a few species cause disease in humans [3]. The incidence and prevalence of NTM pulmonary disease (NTM-PD) are increasing in many countries [4–18]. In addition, NTM-PD is difficult to diagnose and requires long-term treatment, and there are many treatment failures or reinfections [1, 2]. The burden of medical expenses is also high [1]. Therefore, NTM-PD is becoming an emerging global threat worldwide.

Most of the epidemiology of NTM-PD has been identified using laboratory-based surveillance and claims data. An analysis of NTM patients isolated at the reference laboratory of mycobacteriology in Denmark confirmed that the prevalence of definitive NTM disease was 1.20 cases/100,000 population, with no significant change over 25 years [19]. In Ontario, Canada, a mycobacteria laboratory reported an increase in NTM disease from 4.65 cases/100,000 population in 1998 to 9.08 cases/100,000 population in 2010 [12]. Population-based studies of NTM-PD using claims data have been conducted in several countries and have identified an increasing global burden on NTM-PD [6, 11, 14, 16–18, 20]. In the United States, the claims-based incidence was 4.7/100,000 in the population, and the prevalence was 11.7/100,000 in 2015 after analyzing data from 27 million people [14]. The annual claims-based incidence and prevalence increased by 5.2% and 7.5%, respectively [14].

The epidemiology of NTM in Korea was estimated from claims data, and the incidence and prevalence of NTM-PD were also confirmed to have increased [16–18]. Lee et al. analyzed the Health Insurance Review & Assessment Service (HIRA) data from 2007 to 2016 and reported that the prevalence of NTM-PD rose from 6.7 cases/100,000 population to 39.6 cases/100,000 population, and the incidence increased from 6.0 cases/100,000 population to 19 cases/100,000 population [17]. Kim et al. found that only 23.4% of NTM patients had received complex treatment within one year, and 18.8% of them had received macrolide complex treatment [16]. An increase in NTM-PD patients was also confirmed in a study at a tertiary hospital in Korea [21].

With the increase in NTM-PD, the importance is increasing, and diagnostic tests are essential for the diagnosis of NTM-PD [22, 23]. Unlike tuberculosis, patients who are diagnosed with NTM-PD alone do not require treatment [22, 23]. Patients with suspected NTM-PD who do not meet the diagnostic criteria should continue to be followed up until the diagnosis is confirmed or excluded [23]. Since the treatment regimen for NTM is different depending on the causative organism, empirical treatment should not be started without identifying the causative organism [23]. Because the drug susceptibilities for the *M. abscessus* complex are variable, drug susceptibility tests (DSTs) should be performed on all diagnosed NTM patients [23].

The studies of NTM-PD epidemiology conducted with the claims data were performed based on the diagnostic codes [6, 11, 14, 16–18, 20]. The results of an in-depth analysis of laboratory tests and treatment drugs related to NTM-PD have not yet been reported worldwide. Therefore, this study aims to identify the status of laboratory tests and treatments related to NTM-PD in Korea.

## Methods

### HIRA big data

Korea has an NHI that covers 98% of the total population, numbering approximately 50 million as of 2014 [24]. HIRA big data are generated while reimbursing health care providers under the NHI, which is an obligation for all medical institutes. The data include comprehensive information about age, gender, diagnosis, hospitalization, outpatient service, procedures, surgeries, examination, treatment, prescriptions, and cost [24–26]. The database has been accessible to all researchers since 2009 [24].

### Study population

Patients with a diagnostic code of NTM (A31, infection due to other mycobacteria) according to the International Classification of Disease-Tenth Revision (ICD-10) coding system were retrospectively identified between January 1st, 2007, and June 30th, 2019 from the HIRA main database. The relevant information was extracted from secondary accessible research data. This study used research data (M20200229346) from the HIRA database. The views expressed are those of the authors and not necessarily those of the HIRA and the Ministry of Health and Welfare (MOHW).

### Definition of NTM-PD

Diagnostic code-based NTM-PD patients were defined as patients who had NTM (ICD-10: A31) as a primary or secondary diagnosis on at least 2 occasions within 180 days. Cutaneous mycobacterial infection (ICD-10: A31.1) was excluded as a diagnosis of NTM. The Korean Classification of Diseases (KCD), similar to ICD-10, did not include disseminated *Mycobacterium avium-intracellulare* complex (ICD-10: A31.2); thus, excluding this condition was not required. The first year, 2007, was used as a washout period, and patients from 2008 to 2018 were analyzed. The incidence and prevalence were calculated for the population according to sex and age groups with data from the Ministry of the Interior and Safety (https://jumin.mois.go.kr/). Clinically refined NTM-PD patients were excluded from diagnostic code-based NTM-PD patients diagnosed at medical institutions with an implementation rate of less than 5% of diagnosis-related AFB cultures. For a comprehensive patient definition, clinically refined NTM-PD patients were identified by excluding medical institutions with low AFB culture rates and then reanalyzing the data according to the criteria for diagnostic code-based NTM-PD patients.

### NTM-related laboratory tests

NTM-related laboratory tests, including acid-fast bacilli (AFB) smear, AFB culture, AFB DST, and NTM identification, were analyzed. In Korea, medical institutions can request laboratory tests from commercial laboratories regardless of whether they have a laboratory, and the doctor who ordered the test can check the results without delay on the website. Diagnosis-related AFB staining and AFB cultures associated with the diagnosis were included from 120 days before the diagnosis to 30 days after the diagnosis. NTM identification and drug susceptibility tests were included 60 days before the diagnosis and 180 days after the diagnosis. The diagnostic tests included requests for a specific period from all medical institutions, not only those that claimed the diagnosis of NTM-PD. These criteria included a duration that was twice the previous durations for evaluating the adequacy of treatment for tuberculosis in Korea [27]. The methods of NTM identification included nested PCR, PCR hybridization, and PCR restriction fragment length polymorphisms covered by the NHI but did not contain the sequencing method.

### Statistical analysis

Statistical analysis was performed using R project (version 3.5.1). Categorical values are presented as frequencies, and continuous values are presented as medians and interquartile ranges (IQRs). For comparing continuous values, the Mann–Whitney U test and Kruskal–Wallis test were used. In the pairwise Wilcoxon rank sum test, the *P* value was adjusted by the Bonferroni method. The 95% confidence intervals (CIs) of incidence and prevalence were calculated by normal approximation**.** The annual percentage increase was estimated using the negative binomial regression model.

## Results

### Newly diagnosed NTM-PD patients

**Fig. 1:**
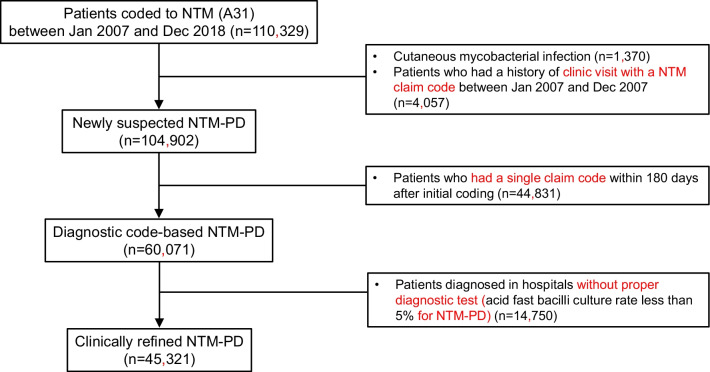
Flow chart to define clinically refined NTM-PD patients. NTM, nontuberculous mycobacteria; NTM-PD, nontuberculous mycobacterial pulmonary disease

Figure 1 summarizes the process of defining the NTM-PD patients. From January 2007 to December 2018, a total of 110,329 patients were treated for NTM. Among them, 1370 patients had cutaneous mycobacterial infections, and 4057 patients were treated in 2007. Of the 104,902 patients that were included after excluding the above patients, 60,071 patients were estimated to have NTM-PD, except for 44,831 patients who did not visit hospitals within six months or who returned after six months.

### AFB culture rate by medical institution

**Fig. 2:**
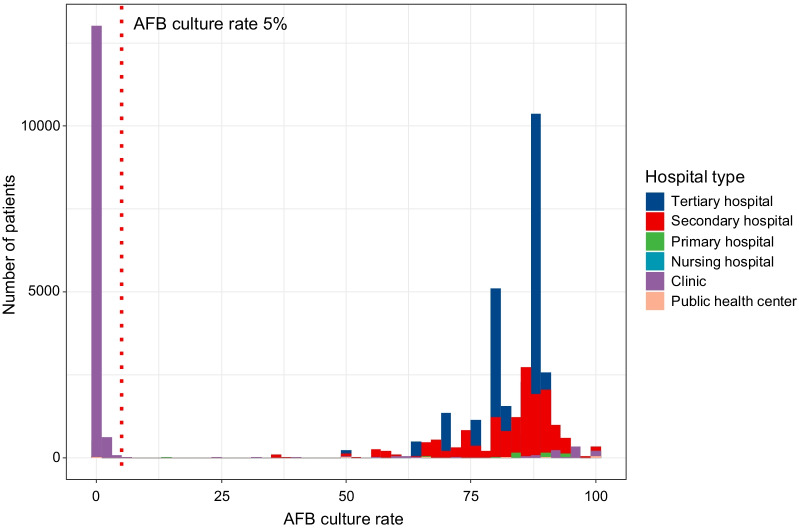
Histogram of the AFB culture rate in newly diagnosed NTM-PD patients according to hospital. AFB, acid-fast bacilli; NTM-PD, nontuberculous mycobacterial pulmonary disease

Figure 2 presents a histogram of the AFB culture rate of the diagnostic code-based NTM-PD patients by each medical institution. The median AFB culture rate was 85.0% (IQR 81.4–89.7%) in tertiary hospitals that diagnosed 28,547 (44.7%) patients, 86.8% (IQR 73.6–100.0%) in secondary hospitals that diagnosed 17,237 (27.0%) patients, 0.0% (IQR 0.0–91.3%) in clinics that diagnosed 16,397 (25.6%) patients, 58.7% (IQR 0.0–100%) in primary hospitals that diagnosed 1490 (2.3%) patients, 100.0% (IQR 50.0–100%) in public health centers that diagnosed 169 (0.3%) patients, and 100.0% (IQR 50.0–100.0%) in nursing hospitals that diagnosed 92 (0.1%) patients. The AFB culture rates by medical institution showed no significant difference between the tertiary hospitals and secondary hospitals, the tertiary hospitals and primary hospitals, and the secondary hospitals and primary hospitals (*P* value > 0.999, > 0.999, = 0.601, respectively). However, the culture rates of the clinics were significantly different from those of the tertiary hospitals, secondary hospitals, public health centers, and nursing hospitals (*P* value < 0.001). Therefore, institutions with an AFB culture rate of less than 5% in diagnostic code-based NTM-PD patients were considered inaccurate and were excluded from the clinically refined NTM-PD patients. To define patients comprehensively, medical institutions without proper tests were excluded, instead of individual patient-specific criteria. As a result, 41.1% (553/1345) of the medical institutions and 14,750 (24.6%) patients were excluded. The patients that were excluded based on the type of medical institution were 13,683 (92.8%) clinic patients, 510 (3.5%) primary hospital patients, 481 (3.3%) tertiary hospital patients, 40 (0.3%) secondary hospital patients, 23 (0.1%) public health center patients, and 13 (0.1%) nursing hospital patients.

### Characteristics of the clinically refined NTM-PD patients

Table 1 summarizes the characteristics of the clinically refined NTM-PD patients based on the diagnostic code and the AFB culture rate. After reflecting the AFB culture rate, clinically refined NTM-PD patients included 19,551 male (43.1%) and 25,770 female (56.9%) patients. The number of patients by age group was 11,994 (26.5%) patients in the 60–69 age group, 11,308 (25.0%) patients in the 70–79 age group, and 10,522 (23.2%) patients in the 50–59 age group. When comparing the changes after applying the AFB culture rates, the number of female patients decreased from 38,092 (63.4%) to 25,770 (56.9%). The proportion of patients under age 50 decreased after recalculation. According to the type of hospital, the number of patients in the clinic decreased from 14,998 (25.6%) to 909 (2.0%). However, the number of patients in tertiary hospitals and secondary hospitals did not decrease significantly.

**Table 1:** Characteristics of nontuberculous mycobacterial pulmonary disease (NTM-PD) patients

Features	Diagnostic code-based NTM-PD	Clinically refined NTM-PD
*Sex*		
Male	21,979 (36.6)	19,551 (43.1)
Female	38,092 (63.4)	25,770 (56.9)
*Age group*		
–19	1685 (2.8)	471 (1.0)
20–29	3797 (6.3)	713 (1.6)
30–39	5136 (8.5)	1673 (3.7)
40–49	7536 (12.5)	4436 (9.8)
50–59	12,786 (21.3)	10,522 (23.2)
60–69	12,935 (21.5)	11,994 (26.5)
70–79	11,808 (19.7)	11,308 (25.0)
80–	4388 (7.3)	4204 (9.3)
*Insurance type*		
Medical insurance	56,981 (94.9)	42,704 (94.2)
Medical aid	3018 (5.0)	2544 (5.6)
Veterans’ health care	72 (0.1)	73 (0.2)
*Hospital type*		
Tertiary hospital	27,253 (45.4)	26,772 (59.1)
Secondary hospital	16,159 (26.9)	16,119 (35.6)
Hospital	1419 (2.4)	909 (2.0)
Nursing hospital	85 (0.1)	72 (0.2)
Clinic	14,998 (25.0)	1315 (2.9)
Public health center	157 (0.2)	134 (0.3)
Total	60,071	45,321

### Annual incidence and prevalence of NTM-PD

**Fig. 3:**
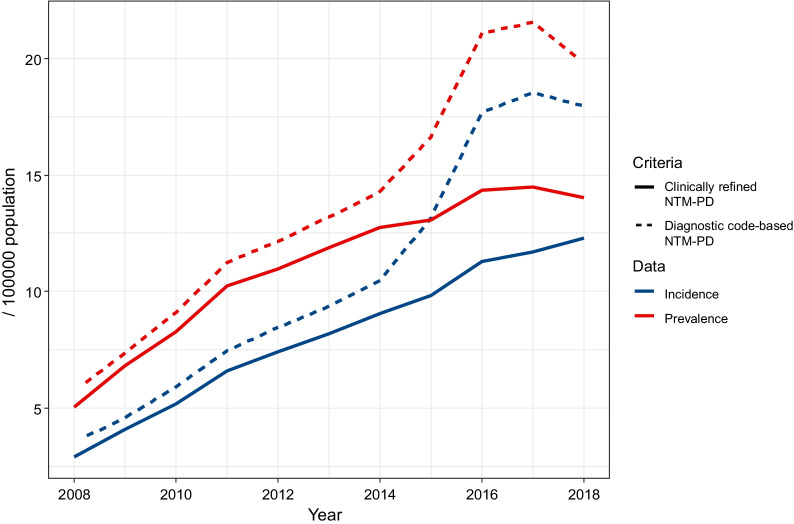
Annual incidence and prevalence of newly diagnosed NTM-PD patients. NTM-PD = nontuberculous mycobacterial pulmonary disease

The annual incidence and prevalence according to diagnostic code-based and clinically refined NTM-PD are presented in Table 2 and Additional file 1: Table S1. The incidence of diagnostic code-based NTM-PD per 100,000 population was 3.5 (3.4–3.7) in 2008, 5.9 (5.7–6.1) in 2010, 8.4 (8.2–8.7) in 2012, 10.5 (10.2–10.7) in 2014, 17.7 (17.3–18.0) in 2016 and 18.0 (17.6–18.3) in 2018. The increase in incidence was significant from 2014 to 2016. The annual increase in the rate that was estimated by negative binomial regression was 16.7% (95% CI 14.6–18.9%). After excluding the medical institutions with low AFB culture rates, the incidence of clinically refined NTM-PD cases per 100,000 population was 2.9 (2.8–3.1) in 2008, 5.2 (5.0–5.4) in 2010, 7.4 (7.2–7.6) in 2012, 9.0 (8.8–9.3) in 2014, 11.3 (11.0–11.6) in 2016, and 12.3 (12.0–12.6) in 2018. The annual increase in the rate as estimated by negative binomial regression was 13.2% (95% CI 10.6–16.0%). Depending on the patient definition, the annual incidence varied by approximately 10%. According to the definition, the difference has recently become noticeable, and the incidence decreased by 25.5% (13.2 → 9.8) in 2015 and 36.8% (18.5 → 11.7) in 2017 (Fig. 3).

**Table 2:** Annual incidence of nontuberculous mycobacterial pulmonary disease (NTM-PD) in Korea

Year	Population			Diagnostic code-based NTM-PD						Clinically refined NTM-PD					
				Cases			Incidence			Cases			Incidence		
	Male	Female	Total	Male	Female	Total	Male	Female	Total	Male	Female	Total	Male	Female	Total
2008	24,822,897	24,717,470	49,540,367	831	917	1748	3.3 (3.1–3.6)	3.7 (3.5–4)	3.5 (3.4–3.7)	682	759	1441	2.7 (2.5–3)	3.1 (2.9–3.3)	2.9 (2.8–3.1)
2009	24,929,939	24,843,206	49,773,145	986	1281	2267	4.0 (3.8–4.3)	5.2 (4.9–5.4)	4.6 (4.4–4.7)	888	1135	2023	3.6 (3.3–3.8)	4.6 (4.3–4.8)	4.1 (3.9–4.2)
2010	25,310,385	25,205,281	50,515,666	1255	1722	2977	5.0 (4.7–5.2)	6.8 (6.5–7.2)	5.9 (5.7–6.1)	1147	1465	2612	4.5 (4.3–4.8)	5.8 (5.5–6.1)	5.2 (5.0–5.4)
2011	25,406,934	25,327,350	50,734,284	1636	2156	3792	6.4 (6.1–6.8)	8.5 (8.2–8.9)	7.5 (7.2–7.7)	1497	1840	3337	5.9 (5.6–6.2)	7.3 (6.9–7.6)	6.6 (6.4–6.8)
2012	25,504,060	25,444,212	50,948,272	1851	2447	4298	7.3 (6.9–7.6)	9.6 (9.2–10)	8.4 (8.2–8.7)	1686	2084	3770	6.6 (6.3–6.9)	8.2 (7.8–8.5)	7.4 (7.2–7.6)
2013	25,588,336	25,553,127	51,141,463	2015	2786	4801	7.9 (7.5–8.2)	10.9 (10.5–11.3)	9.4 (9.1–9.7)	1852	2333	4185	7.2 (6.9–7.6)	9.1 (8.8–9.5)	8.2 (7.9–8.4)
2014	25,669,296	25,658,620	51,327,916	2170	3197	5367	8.5 (8.1–8.8)	12.5 (12–12.9)	10.5 (10.2–10.7)	1972	2672	4644	7.7 (7.3–8)	10.4 (10–10.8)	9.0 (8.8–9.3)
2015	25,758,186	25,771,152	51,529,338	2547	4235	6782	9.9 (9.5–10.3)	16.4 (15.9–16.9)	13.2 (12.8–13.5)	2185	2869	5054	8.5 (8.1–8.8)	11.1 (10.7–11.5)	9.8 (9.5–10.1)
2016	25,827,594	25,868,622	51,696,216	2924	6214	9138	11.3 (10.9–11.7)	24.0 (23.4–24.6)	17.7 (17.3–18.0)	2544	3278	5822	9.8 (9.5–10.2)	12.7 (12.2–13.1)	11.3 (11.0–11.6)
2017	25,855,919	25,922,625	51,778,544	2784	6805	9589	10.8 (10.4–11.2)	26.3 (25.6–26.9)	18.5 (18.1–18.9)	2482	3579	6061	9.6 (9.2–10)	13.8 (13.4–14.3)	11.7 (11.4–12)
2018	25,866,129	25,959,930	51,826,059	2980	6332	9312	11.5 (11.1–11.9)	24.4 (23.8–25)	18.0 (17.6–18.3)	2616	3756	6372	10.1 (9.7–10.5)	14.5 (14.0–14.9)	12.3 (12.0–12.6)
Overall				21,979	38,092	60,071	7.8 (7.7–7.9)	13.6 (13.5–13.7)	10.7 (10.6–10.8)	19,551	25,770	45,321	7.0 (6.9–7.1)	9.2 (9.1–9.3)	8.1 (8.0–8.2)

### Incidence of NTM-PD by sex and age groups

**Fig. 4:**
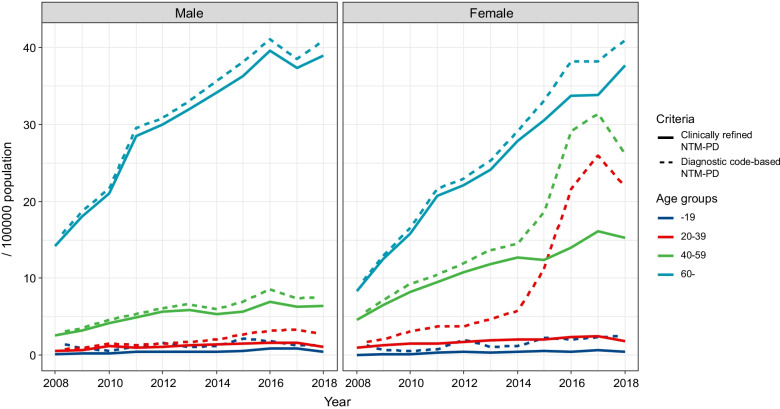
Annual incidence of newly diagnosed NTM-PD patients stratified by sex and age groups. NTM-PD = nontuberculous mycobacterial pulmonary disease

Figure 4 shows the annual incidence of NTM-PD patients by sex and age groups. The incidence in male patients did not show a significant change according to the definition. The difference in incidence according to the criteria was noteworthy in female patients aged 20–39 years and 40–59 years. These patients were mainly diagnosed in clinics and primary hospitals. The number of patients aged 20–39 years decreased by 1.2% and 0.2% in tertiary and secondary hospitals, respectively, but decreased by 96.3% in primary hospitals and 98.8% in clinics. Similarly, patients aged 40–59 years decreased by 1.7% and 0.3% in tertiary and secondary hospitals, respectively, but decreased by 58.8% in primary hospitals and 94.6% in clinics.

A detailed analysis of the incidence of NTM-PD in female patients aged 20–59 years was performed (Additional file 1: Table S2). Since 2016, the diagnostic code-based incidence of the patients who are 20–49 years of age has increased rapidly. Comparing the diagnostic code-based NTM-PD and the clinically refined NTM-PD incidence per 100,000 population in 2017, there was a 93.0% (26.4 → 1.86) decrease in the 20–29 year-old patients, an 87.9% (25.6 → 3.09) decrease in the 30–39 year-old patients, and a 63.1% (25.4 → 9.36) decrease in the 40–49 year-old patients.

### Laboratory test rate in the clinically refined NTM-PD patients by hospital type

Among the 45,321 clinically refined NTM-PD patients, laboratory tests were performed, AFB staining was performed in 35,001 (77.2%) patients, 38,220 (84.3%) patients had an AFB culture, 26,772 (59.1%) patients had NTM identification, and 18,289 (40.4%) patients had AFB DST (Table 3). The median test rates of tertiary hospitals were 78.2% for AFB staining, 85.0% for AFB culture, 62.4% for NTM identification, and 42.8% for AFB DST. The median test rates of clinics were 69.3% for AFB staining, 82.9% for AFB culture, 43.7% for NTM identification, and 22.5% for AFB DST. When the AFB culture rate of each medical institution was compared with that of tertiary hospitals, it was similar or higher in all of the medical institution types. Widening the acceptable time window for microbiological results to between 180 days before and 180 days after the diagnosis altered the proportions with 82.6% for AFB smear, 89.7% for AFB culture, 65.4% for NTM identification, and 45.7% for AFB DST. These differences were around 5–10%, confirming that the initial criteria were acceptable.

**Table 3:** Nontuberculous mycobacteria (NTM)-related laboratory test rate according to hospital type

Hospital type	No. of patients	No. of hospitals	AFB smear (%)	AFB culture (%)	NTM identification (%)	DST (%)
Tertiary hospital	26,772	54	78.2 (69.6, 82.0, 88.3)	85.0 (81.6, 84.9, 89.3)	62.4 (53.1, 61.2, 68.3)	42.8 (25.1, 39.6, 53.1)
Secondary hospital	16,119	288	75.8 (69.4, 85.0, 100)	83.2 (75.0, 87.4, 100)	55.6 (41.3, 60.0, 75.0)	38.2 (16.6, 37.5, 56.3)
Hospital	909	169	87.6 (90.0, 100, 100)	85.8 (90.0, 100, 100)	49.0 (0.0, 58.6, 100.0)	37.1 (0.0, 33.3, 100)
Nursing hospital	72	65	83.3 (100, 100, 100)	95.8 (100, 100, 100)	43.1 (0, 0, 100)	20.8 (0, 0, 0)
Clinic	1315	177	69.3 (85.7, 100, 100)	82.9 (97.8, 100, 100)	43.7 (0.0, 55.3, 100)	22.5 (0, 10.0, 100.0)
Public health center	134	58	73.1 (50.0, 100, 100)	82.8 (76.2, 100, 100)	38.8 (0, 43.2, 66.7)	26.9 (0, 0, 44.5)
Total	45,321	778	77.2	84.3	59.1	40.4

AFB, acid-fast bacilli; DST, drug susceptibility test

The values in parentheses are the median and quantiles of the test rates for each hospital

### Medical usage behavior of the clinically refined NTM-PD patients

A total of 784,792 claims of NTM-PD patients were analyzed for the NTM patients' medical usage when NTM was the primary or the secondary diagnosis. There were 27,546 inpatient claims and 757,246 outpatient claims. In the outpatient setting, the median number of visits to medical institutions by a patient was 9 (IQR 4–19), and the median interval was 31.5 (IQR 15.5–58) days (Table 4). The median duration from the initial visit to the last visit was 448 (IQR 114–1167) days. The median number of NTM-related visits was 8 (IQR 4–18) for men and 9 (IQR 4–20) for women, and there was a significant difference between them (*P* value < 0.001). The median number of NTM-related visits was 9 (IQR 4–19) for medical insurance patients and 9 (IQR 4–20) for medical aid patients, and there was no significant difference between them (*P* value > 0.999).

**Table 4:** Medical usage statistics of nontuberculous mycobacterial pulmonary disease patients

Features	Number of visits	Median interval of visits (days)	Total follow-up duration (days)
*Sex*			
Male	8 (4–18)	31 (15–56)	198 (102–1011)
Female	9 (4–20)	33 (16–60)	490 (125–1287)
*Insurance type*			
Medical insurance	9 (4–19)	32 (16–59)	452 (117–1182)
Medical aid	9 (4–20)	29 (16–50)	378 (95–966)
Veterans’ medical care	14 (4–38)	1 (0–8)	465 (38–1071)
Total	9 (4–19)	31.5 (15.5–58)	448 (114–1167)

## Discussion

In many countries, it has been confirmed that the incidence and prevalence of NTM are increasing nationwide [7–12, 14, 15, 19, 20]. In Korea, an increase in the incidence and prevalence of NTM-PD was also reported using nationwide claims data [16–18]. In this study, the surge of NTM-PD in young women from 2015 was identified as an overestimation without evidence from the proper laboratory tests.

The epidemiology of NTM-PD has been reported mainly in developed countries. During the evaluation of the epidemiology of NTM-PD, the study, which defined culture-confirmed NTM, is the most accurate [15, 19]. This is only possible in countries where NTM-PD is a notifiable disease, such as in Australia [15], or where most mycobacterial tests are performed by national reference laboratories, such as in Denmark [19]. Estimating the epidemiology of NTM-PD using claims data is highly representative [11, 14, 16–18, 20]. Some studies have used multiple claims for NTM to increase the specificity or have estimated the epidemiology of NTM-PD when patients are treated with antimycobacterial agents [14, 16, 20]. We conservatively measured the epidemiology of NTM-PD. The newly diagnosed NTM-PD patients were identified with multiple claims for NTM within six months. Furthermore, outliers were excluded using the diagnosis related to the AFB culture rates of the medical institutions. The AFB culture is a necessary test to distinguish MTB from NTM and to perform subsequent identification and DST.

In Korea, the incidence and prevalence of tuberculosis are decreasing due to continuous tuberculosis management, but the incidence and prevalence of NTM-PD are increasing [28]. Although the epidemiology varies by the definition used, the incidence per 100,000 population of NTM-PD in Korea was reported as 17.9–26.6 cases in 2016 [16–18]. In this study, after removing outliers, it decreased from 17.7 cases to 11.3 cases, which showed a significant difference from previous reports. This change was prominent in women, where the incidence per 100,000 population in 2016 was 25.0–32.0 cases [16, 18], but in this study, it was reduced by nearly half to 12.7 cases. Lee et al. also included the AFB culture in the NTM-PD definition without the difference between the diagnosis date and the test date [17]. NTM-PD patients can be defined by the individual patient's diagnostic code and the AFB culture results, but the long time required for examination and revisits may result in unnecessary patient exclusion. According to the microbiologic criteria of NTM-PD, sputum culture should be positive at least twice [22, 23], but the test results were unavailable from the claims data. Therefore, to remove significant outliers, the reliability of NTM-PD diagnosis was determined by the AFB culture rate of each hospital. The criterion of 5% is considered a low number, but the distribution by medical institution showed a significant difference, so even if the criterion was higher, the difference was negligible.

Most of the NTM-PD patients without AFB culture were women in the 20–39 and 40–59 age groups, and they were diagnosed at the clinics. The number of clinics that diagnosed patients with NTM-PD without AFB culture was biased toward some of the data, but a detailed analysis was impossible due to HIRA's policy. Given that macrolides used in the treatment of NTM-PD are also used in the treatment of sexually transmitted diseases, many of these NTM diagnostic codes may have been used to prescribe antimicrobial agents in connection with insurance and privacy issues. Using a diagnostic code with a high reimbursement and considering profit-driven motivation has already been pointed out as a limitation of the claims data [24].

Various studies using claims data are being conducted, and studies to estimate the prevalence and incidence of diseases are easily performed. However, in many cases, only the diagnostic codes are used. Although it varies according to each country's medical insurance policy, these claims data are accurate for diseases such as cancer or rare diseases directly related to insurance coverage in Korea [24, 25]. For infectious diseases such as tuberculosis, which are government-controlled and funded, the diagnostic or insurance codes are reliable in Korea [27, 29]. It was pointed out that caution is needed in using the diagnostic code in the claim data as it is due to its inherent nature [24, 25]. This remains a limitation for most studies of NTM-PD using claims data so far [6, 11, 14, 16–18, 20]. Recently, the validity of code-based claims of NTM-PD in patients with bronchiectasis in the United States was confirmed [30]. However, this applies to patients with risk factors, and in the case of the general population, the diagnostic code alone may be inaccurate, as in this study. The method will vary depending on the disease but including the information of whether appropriate laboratory tests have been performed can improve the accuracy of the epidemiology.

NTM-PD is generally known to be more prevalent in women [1, 2], but the prevalence varies from country to country. After removing the outliers in this study, the proportion of women in our cohort decreased from 63.4 to 56.9%. A tertiary hospital in Korea reported that 59.8% of NTM-PD patients were female [21], which was similar to our results. In Germany, it has been reported that NTM-PD is not more prevalent in women [11].

NTM-PD patients were mainly treated at tertiary and secondary hospitals in Korea. The laboratory test rates of tertiary hospitals were higher than those of other medical institutions. To provide a proper examination, management of the underlying disease, and long-term treatment, treatment at a higher-level medical institution was carried out. In Germany, pulmonologists gave patients a longer treatment period and had a higher guideline-based therapy rate for NTM-PD than general practitioners [31]. It is important to continuously follow NTM-PD patients. In this study, the number of hospital visits for NTM-PD did not differ between health-insured and medical aid patients. The median observation period for treated patients was 11 months. It is recommended to continue NTM-PD treatment for 12 months after culture conversion [22, 23], but it has been confirmed that most of the patients were not adequately treated. Further analysis is needed on the treatment pattern of NTM-PD patients.

The regional distribution of NTM-PD could not be assessed in this study. Due to the nature of the HIRA data, information on the location of the medical institutions is provided. Lee et al. reported that the NTM incidence was high in Seoul and Gyeonggi provinces, which are northern regions of Korea, using the HIRA data [17]. As NTM is known to be more prevalent in tropical areas [1], it was the opposite result. Because the major tertiary hospitals are in the capital, Seoul, and because patients living in rural areas can visit and return to hospitals in Seoul within a day by train or bus, the incidence rate in Seoul and surrounding areas seems to have risen. Therefore, the incidence report according to the location of medical institutions by Lee et al. is an inappropriate analysis in Korea [17]. In Japan, the incidence of NTM increased as the latitude decreased [13].

There are several limitations in this study. First, the NTM-PD definition according to the American Thoracic Society and Infectious Diseases Society of America guideline was not applicable. The lack of diagnostic test results, such as AFB culture, chest X-ray, and chest computed tomography, is a fundamental limitation of claims data [6, 11, 14, 16–18, 20]. There are other methods to evaluate medical big data, such as the common data model. However, data from some participating hospitals are available, and microbiological test results have not yet been included in Korea [32]. Second, the AFB culture rate by each hospital was applied for accurate diagnosis, but this is not a definition for individual patients. However, we used this criterion for a comprehensive patient definition suitable for big data analysis, such as when a misdiagnosed patient is later diagnosed with an appropriate test at another hospital. Third, subgroup analysis according to the immune status was not performed, but it is not known as an important underlying condition in Korean NTM-PD [17]. Fourth, data from the HIRA only include insurance-covered medical practices. A few tertiary hospitals using uninsured sequence analysis for NTM identification were excluded from the statistics. Therefore, the NTM identification rate is underestimated in this study.

## Conclusions

This study improved the accuracy of the estimates of incidence and prevalence by evaluating the laboratory tests that are essential for diagnosing NTM-PD. The incidence of NTM in Korea is increasing, but the apparent surge in cases among young women cannot be substantiated. When conducting claim-based research, additional information should always be considered for inclusion.

## Supplementary Information

**Additional file 1: Table S1**. Annual prevalence of nontuberculous mycobacterial pulmonary disease (NTM-PD) in Korea. **Table S2**. Annual incidence of nontuberculous mycobacterial pulmonary disease (NTM-PD) in female patients in the 20–59 age group (% difference).

## Data Availability

The data that support the findings of this study are available from the Health Insurance Review & Assessment Service in Korea, but restrictions apply to the availability of these data, which were used under license for the current study, and so are not publicly available.

## Abbreviations

AFB: Acid-fast bacilli
CI: Confidence interval
DST: Drug susceptibility test
HIRA: Health Insurance Review and Assessment Service
ICD-10: International Classification of Disease-Tenth Revision
IQR: Interquartile ranges
MTB: *Mycobacterium tuberculosis* Complex
NHI: National Health Insurance Service
NTM: Nontuberculous mycobacteria
NTM-PD: Nontuberculous mycobacteria pulmonary disease
