# Supplementary material for: Clinically refined epidemiology of nontuberculous mycobacterial pulmonary disease in South Korea: overestimation when relying only on diagnostic codes
Source: BMC Pulm Med. 2022 May 13;22:195. doi: 10.1186/s12890-022-01993-1 (PMC9107265; doi:10.1186/s12890-022-01993-1)
Supplement: Supplementary file 1 — Additional file 1: Table S1. Annual prevalence of nontuberculous mycobacterial pulmonary disease (NTM-PD) in Korea. Table S2. Annual incidence of nontuberculous mycobacterial pulmonary disease (NTM-PD) in female patients in the 20–59 age group (% difference). [file 12890_2022_1993_MOESM1_ESM.docx]

Table S1. Annual prevalence of nontuberculous mycobacterial pulmonary disease (NTM-PD) in Korea

| Year | Population | | |  | Diagnostic code-based NTM-PD | | | | | |  | Clinically refined NTM-PD | | | | | |
| --- | --- | --- | --- | --- | --- | --- | --- | --- | --- | --- | --- | --- | --- | --- | --- | --- | --- |
|  |  |  |  |  | Cases | | | Prevalence | | |  | Cases | | | Prevalence | | |
|  | Male | Female | Total |  | Male | Female | Total | Male | Female | Total |  | Male | Female | Total | Male | Female | Total |
| 2008 | 24822897 | 24717470 | 49540367 |  | 1271 | 1563 | 2834 | 5.1  (4.8-5.4) | 6.3  (6.0-6.6) | 5.7  (5.5-5.9) |  | 1108 | 1394 | 2502 | 4.5  (4.2-4.7) | 5.6  (5.3-5.9) | 5.1  (4.9-5.2) |
| 2009 | 24929939 | 24843206 | 49773145 |  | 1503 | 2161 | 3664 | 6.0  (5.7-6.3) | 8.7  (8.3-9.1) | 7.4  (7.1-7.6) |  | 1385 | 1997 | 3382 | 5.6  (5.3-5.8) | 8.0  (7.7-8.4) | 6.8  (6.6-7.0) |
| 2010 | 25310385 | 25205281 | 50515666 |  | 1876 | 2723 | 4599 | 7.4  (7.1-7.7) | 10.8  (10.4-11.2) | 9.1  (8.8-9.4) |  | 1749 | 2435 | 4184 | 6.9  (6.6-7.2) | 9.7  (9.3-10.0) | 8.3  (8.0-8.5) |
| 2011 | 25406934 | 25327350 | 50734284 |  | 2384 | 3327 | 5711 | 9.4  (9.0-9.8) | 13.1  (12.7-13.6) | 11.3  (11.0-11.5) |  | 2218 | 2978 | 5196 | 8.7  (8.4-9.1) | 11.8  (11.3-12.2) | 10.2  (10.0-10.5) |
| 2012 | 25504060 | 25444212 | 50948272 |  | 2624 | 3559 | 6183 | 10.3  (9.9-10.7) | 14.0  (13.5-14.4) | 12.1  (11.8-12.4) |  | 2447 | 3146 | 5593 | 9.6  (9.2-10) | 12.4  (11.9-12.8) | 11.0  (10.7-11.3) |
| 2013 | 25588336 | 25553127 | 51141463 |  | 2810 | 3937 | 6747 | 11.0  (10.6-11.4) | 15.4  (14.9-15.9) | 13.2  (12.9-13.5) |  | 2626 | 3449 | 6075 | 10.3  (9.9-10.7) | 13.5  (13.0-13.9) | 11.9  (11.6-12.2) |
| 2014 | 25669296 | 25658620 | 51327916 |  | 2956 | 4374 | 7330 | 11.5  (11.1-11.9) | 17.0  (16.5-17.6) | 14.3  (14.0-14.6) |  | 2746 | 3798 | 6544 | 10.7  (10.3-11.1) | 14.8  (14.3-15.3) | 12.7  (12.4-13.1) |
| 2015 | 25758186 | 25771152 | 51529338 |  | 3259 | 5318 | 8577 | 12.7  (12.2-13.1) | 20.6  (20.1-21.2) | 16.6  (16.3-17.0) |  | 2878 | 3843 | 6721 | 11.2  (10.8-11.6) | 14.9  (14.4-15.4) | 13.0  (12.7-13.4) |
| 2016 | 25827594 | 25868622 | 51696216 |  | 3625 | 7289 | 10914 | 14.0  (13.6-14.5) | 28.2  (27.5-28.8) | 21.1  (20.7-21.5) |  | 3221 | 4189 | 7410 | 12.5  (12-12.9) | 16.2  (15.7-16.7) | 14.3  (14.0-14.7) |
| 2017 | 25855919 | 25922625 | 51778544 |  | 3380 | 7774 | 11154 | 13.1  (12.6-13.5) | 30.0  (29.3-30.7) | 21.5  (21.1-21.9) |  | 3059 | 4434 | 7493 | 11.8  (11.4-12.3) | 17.1  (16.6-17.6) | 14.5  (14.1-14.8) |
| 2018 | 25866129 | 25959930 | 51826059 |  | 3378 | 6881 | 10259 | 13.1  (12.6-13.5) | 26.5  (25.9-27.1) | 19.8  (19.4-20.2) |  | 3003 | 4255 | 7258 | 11.6  (11.2-12) | 16.4  (15.9-16.9) | 14.0  (13.7-14.3) |
| Overall |  |  |  |  | 29066 | 48906 | 77972 | 10.4  (10.2-10.5) | 17.4  (17.3-17.6) | 13.9  (13.8-14.0) |  | 26440 | 35918 | 62358 | 9.4  (9.3-9.5) | 12.8  (12.7-12.9) | 11.1  (11.0-11.2) |

Table S2. Annual incidence of nontuberculous mycobacterial pulmonary disease (NTM-PD) in female patients in the 20-59 age group (% difference)

|  | Diagnostic code-based NTM-PD | | | |  | Clinically refined NTM-PD | | | |
| --- | --- | --- | --- | --- | --- | --- | --- | --- | --- |
|  | 20-29 | 30-39 | 40-49 | 50-59 |  | 20-29 | 30-39 | 40-49 | 50-59 |
| 2008 | 1.23 | 1.86 | 3.71 | 6.47 |  | 0.54 (-55.8%) | 1.42 (-23.4%) | 3.45 (-7.0%) | 6.21 (-4.0%) |
| 2009 | 1.68 | 2.44 | 4.71 | 10.38 |  | 0.97 (-42.1%) | 1.63 (-33.0%) | 4.10 (-12.9%) | 9.86 (-5.0%) |
| 2010 | 2.67 | 3.39 | 6.26 | 13.08 |  | 0.94 (-64.8%) | 2.00 (-41.0%) | 5.06 (-19.2%) | 12.12 (-7.4%) |
| 2011 | 3.56 | 3.98 | 6.63 | 14.97 |  | 1.15 (-67.8%) | 1.81 (-54.7%) | 5.43 (-18.1%) | 14.25 (-4.8%) |
| 2012 | 3.13 | 4.22 | 7.30 | 17.13 |  | 1.04 (-66.7%) | 2.27 (-46.2%) | 6.13 (-16.1%) | 16.02 (-6.5%) |
| 2013 | 4.40 | 5.02 | 8.29 | 19.65 |  | 1.53 (-65.2%) | 2.19 (-56.3%) | 6.21 (-25.1%) | 18.05 (-8.2%) |
| 2014 | 4.35 | 7.06 | 8.98 | 20.60 |  | 1.27 (-70.8%) | 2.77 (-60.7%) | 6.75 (-24.9%) | 19.05 (-7.5%) |
| 2015 | 9.87 | 12.10 | 14.45 | 22.84 |  | 1.17 (-88.2%) | 2.85 (-76.4%) | 6.89 (-52.3%) | 18.17 (-20.5%) |
| 2016 | 20.32 | 22.65 | 25.07 | 33.40 |  | 1.56 (-92.3%) | 3.15 (-86.1%) | 7.29 (-70.9%) | 20.91 (-37.4%) |
| 2017 | 26.43 | 25.59 | 25.36 | 37.47 |  | 1.86 (-93.0%) | 3.09 (-87.9%) | 9.36 (-63.1%) | 23.12 (-38.3%) |
| 2018 | 24.47 | 19.54 | 21.69 | 30.54 |  | 1.08 (-95.6%) | 2.42 (-87.6%) | 8.63 (-60.2%) | 21.84 (-28.5%) |
| Overall | 9.28 | 9.80 | 12.04 | 20.59 |  | 1.19 (-87.2%) | 2.33 (-76.3%) | 6.30 (-47.7%) | 16.33 (-20.7%) |
